# Secondary cartilage in the murine medial pterygoid plate has a critical role in the pathfinding of the tensor veli palatini

**DOI:** 10.1111/joa.70133

**Published:** 2026-03-15

**Authors:** Luke A. Barlow, Emi Nomura, Abigail S. Tucker

**Affiliations:** ^1^ Centre for Craniofacial and Regenerative Biology, King's College London Guy's Hospital London UK; ^2^ Faculty of Dentistry Institute of Science Tokyo Bunkyo City Tokyo Japan

**Keywords:** endochondral, membranous bone, neural crest, ossification, Runx2, Sox9

## Abstract

The medial pterygoid plate plays a critical role in mammalian craniofacial function during suckling and swallowing. The plate supports the tensor veli palatini (TVP) muscle, which stiffens the soft palate to create a posterior seal. Despite its functional importance, the developmental origins and structural integration of the medial pterygoid plate and associated pterygoid hamulus remain incompletely understood. In this study, we investigated the ontogeny and lineage of the medial pterygoid plate using wildtype and conditional knockout mice with immunofluorescence and lineage tracing. Analysis from embryonic day (E)14.5 to E17.5 confirmed that the medial pterygoid plate formed as a bipartite structure, which later fused to the basisphenoid. The dorsal region of the medial pterygoid plate ossified via Runx2‐dependent intramembranous bone formation, while the ventral region formed as a secondary cartilage, undergoing Sox9‐dependent chondrogenesis, followed by endochondral ossification. The pterygoid hamulus was evident at E14.5 as a condensation of Sox9‐positive mesenchyme at the end of the medial pterygoid plate. Confirming the different modes of development, the ventral and dorsal parts of the medial pterygoid plate showed distinct timing and pattern of collagen remodelling, as shown by B‐CHP. Lineage tracing with *Wnt1Cre;tdTom* and *Mesp1Cre;tdTom* mice demonstrated that the entire pterygoid process formed from neural crest‐derived mesenchyme. In keeping with this, conditional loss of *Runx2* in the neural crest lineage disrupted ossification of the dorsal part of the medial pterygoid plate, whereas conditional loss of *Sox9* abolished chondrogenesis of the ventral part of the medial pterygoid plate and the pterygoid hamulus. Notably, TVP muscle fibres were able to maintain their orientation around the residual cartilage in conditional *Wnt1creRunx2flfl* mutants, while the TVP in conditional *Wnt1creSox9flfl* mutants formed a ball of cells that failed to extend towards the palatal region. The ventral portion of the medial pterygoid plate and hamulus is therefore required to guide early muscle pathfinding. These findings establish the medial pterygoid plate as a compound craniofacial element with distinct ossification modes and an important role interacting and directing neighbouring tissues.

## Graphical abstract

The development of the medial pterygoid plate (MPP) is followed in mouse embryos, highlighting its neural crest origin and complex development via both endochondral and membranous ossification. Using transgenic mice, the secondary cartilage of the MPP is shown to direct the path of the tensor veli palatini.

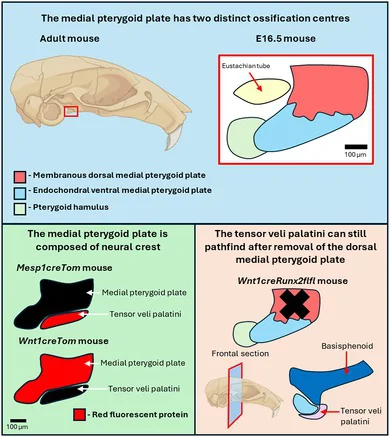

## 1 INTRODUCTION

A defining characteristic of all mammals is their ability to produce milk for their young, facilitating postnatal development before they are capable of independent feeding (Iverson, 2007). For effective suckling, the therian neonate (marsupial and placental mammals) must form both anterior and posterior oral seals to generate the negative intraoral pressure necessary to draw milk into the oral cavity (Crompton, 1995). The posterior oral seal in therian mammals depends on coordinated activity of the tensor veli palatini (TVP), which stiffens the soft palate, and the palatoglossus, mylohyoid, and intrinsic tongue muscles, which raise the posterior portion of the tongue to the soft palate and create a seal (Crompton et al., 2018).

The TVP interacts closely with the pterygoid process positioned on either side of the nasopharynx. The pterygoid process consists of the lateral and medial pterygoid plates, which attach to the basisphenoid component of the sphenoid bone, although the size and morphology of the pterygoid plates vary considerably across mammalian taxa. In mice, the lateral pterygoid plate is rudimentary and instead a lateral pterygoid process has been described, which forms as an extension of the basisphenoid (Fernandez‐Rubio & Radlanski, 2022; Yamamoto et al., 2017, 2021). This murine lateral pterygoid process is not homologous to the lateral pterygoid plate in humans, as it originates as a small cartilaginous extension of the basisphenoid, but it has the same function as an attachment site for the lateral and medial pterygoid muscles (Fernandez‐Rubio & Radlanski, 2022).

The pterygoid hamulus is located at the end of the medial pterygoid plate and provides the bony fulcrum over which the tendon of the TVP redirects before inserting into the palatine aponeurosis (Tachimura et al., 2006). This anatomical configuration is thought to be essential in stiffening the soft palate and thus contributing to the formation of the posterior seal during suckling (Crompton et al., 2018).

The precise relationship between the TVP and the pterygoid hamulus remains unclear. Some sources describe the TVP as gliding around the hamulus before inserting into the soft palate (Koch et al., 1998), while others propose direct insertion onto the hamulus to serve as an anchoring point (Barsoumian et al., 1998). The presence or absence of a bursa—a synovial cushion to reduce friction—is also inconsistently reported (Gray & Goss, 1974). These variations may reflect species‐specific differences, as attachment of the TVP to the hamulus has been observed in primates, pinnipeds, insectivores, and marsupials, but is absent in carnivores and rodents (Edgeworth, 1914). Intraspecific variation also exists; for example, the TVP origin and insertion differ between human ethnic groups (Huang et al., 1997), along with its size being variable between mouse strains (Yamamoto et al., 2020).

In mice, the TVP attaches to the palatine aponeurosis between embryonic day (E)15.5 and E17, coinciding with the onset of jaw movement (Jahan et al., 2010), with the TVP becoming functional between these stages (Nara et al., 2017). The medial pterygoid plate also undergoes rapid morphogenesis during this window (Yamamoto et al., 2017). Although most of the sphenoid bone forms through endochondral ossification, the human pterygoid plates arise from both endochondral and membranous ossification, with the latter involving direct bone formation from mesenchyme (Fawcett, 1910).

In mice, the medial pterygoid plate has been shown to differentiate into two regions after palatal shelf fusion: an upper membranous region with bone spicules and a lower cartilage‐rich region, suggesting it is a compound element formed by both intramembranous and endochondral ossification processes (Yamamoto et al., 2017). Because it forms relatively late compared to other primary cartilages, the medial pterygoid plate has been classified as a secondary cartilage (Hirouchi et al., 2018). Recent work in humans from analysis of histology sections has also shown that the medial pterygoid plate arises from a combination of endochondral and membranous elements, whereas the lateral pterygoid plate derives entirely from membranous bone (Yamamoto et al., 2021).

The murine pterygoid hamulus develops at the end of the medial pterygoid plate, although whether it forms as a separate condensation or an extension of the medial pterygoid remains debated (Yamamoto et al., 2017). In humans, the primordium of the pterygoid hamulus is evident at CS23 and starts to undergo chondrification and synovial bursa formation at 8 weeks gestation (De la Cuadra Bnco et al., 2012). Interestingly, the hamulus was shown to have a distinct chondrification centre compared to the earlier forming medial pterygoid plate (De la Cuadra Bnco et al., 2012).

Anatomically, the pterygoid process lies near the boundary between neural crest‐ and mesoderm‐derived mesenchyme (Evans & Noden, 2006). As such, lineage tracing studies have yielded differing conclusions: McBratney‐Owen et al. (2008) report a neural crest origin for this region, while Shirai et al. (2019) argue for mesodermal contribution. This discrepancy reflects the complexity of craniofacial development and underscores the need for more precise mapping.

In this study, we have built on existing developmental and anatomical research to investigate the structure, origin, and integration of the pterygoid hamulus and medial pterygoid plate and their relationship with the TVP. Through immunofluorescence and lineage tracing in the murine model, we reveal new insights into the ontogeny and significance of this small but functionally critical component of the mammalian skull and show that the ventral medial pterygoid plate is important for muscle pathfinding.

## 2 MATERIALS AND METHODS

### 2.1 Mouse lines

This project used wildtype mice and mutant mouse lines housed in the Biological Services Unit of Guy's Hospital. Breeding of transgenic mouse mutants was approved by the faculty Biological Safety Committee and covered by Home Office licenses, conforming with UK legislation under the Animals (Scientific Procedures) Act 1986 Amendment Regulations (SI 2012/3039). Pregnant dams and embryos were culled using approved schedule 1 methods.

Wildtype (WT) mice were taken at embryonic stage (E) E13.5 to E17.5. *Wnt1Cre;Runx2* mutant samples were generated by crossing *Wnt1Cre; Runx2* ^*fl/+*^ males with *Runx2* ^*fl/fl*^ females to create *Wnt1Cre;Runx2* ^*fl/fl*^ embryos *Wnt1Cre;Sox9* mutant samples were generated by crossing heterozygous *Wnt1Cre; Sox9* ^*fl/+*^ males and *Sox9* ^*fl/fl*^ females to produce *Wnt1Cre;Sox9* ^*fl/fl*^ embryos. Mutant embryos were collected at E14.5, E15.5 and E18.5 (*N* = 3). Cre negative floxed mice were used as littermate controls. To trace the embryonic mesoderm, *Mesp1Cre;tdTom* mice were generated by crossing heterozygous *Mesp1Cre* males with homozygous *tdTom* females to create *Mesp1Cre; tdTom* positive embryos. For the *Wnt1Cre;tdTom* mice, Wnt1Cre/+ males were crossed to homozygous *tdTom* females to produce *Wnt1Cre;tdTom* positive embryos. For both the *Mesp1Cre;tdTom* and *Wnt1Cre;tdTom* lines, E15.5 embryos were taken (*N* = 3). The fluorescence in both lines was confirmed with a NightSea Dual Fluorescent Protein Flashlight (DFP1).

### 2.2 Tissue preparation for staining and immunofluorescence

**FIGURE 1:**
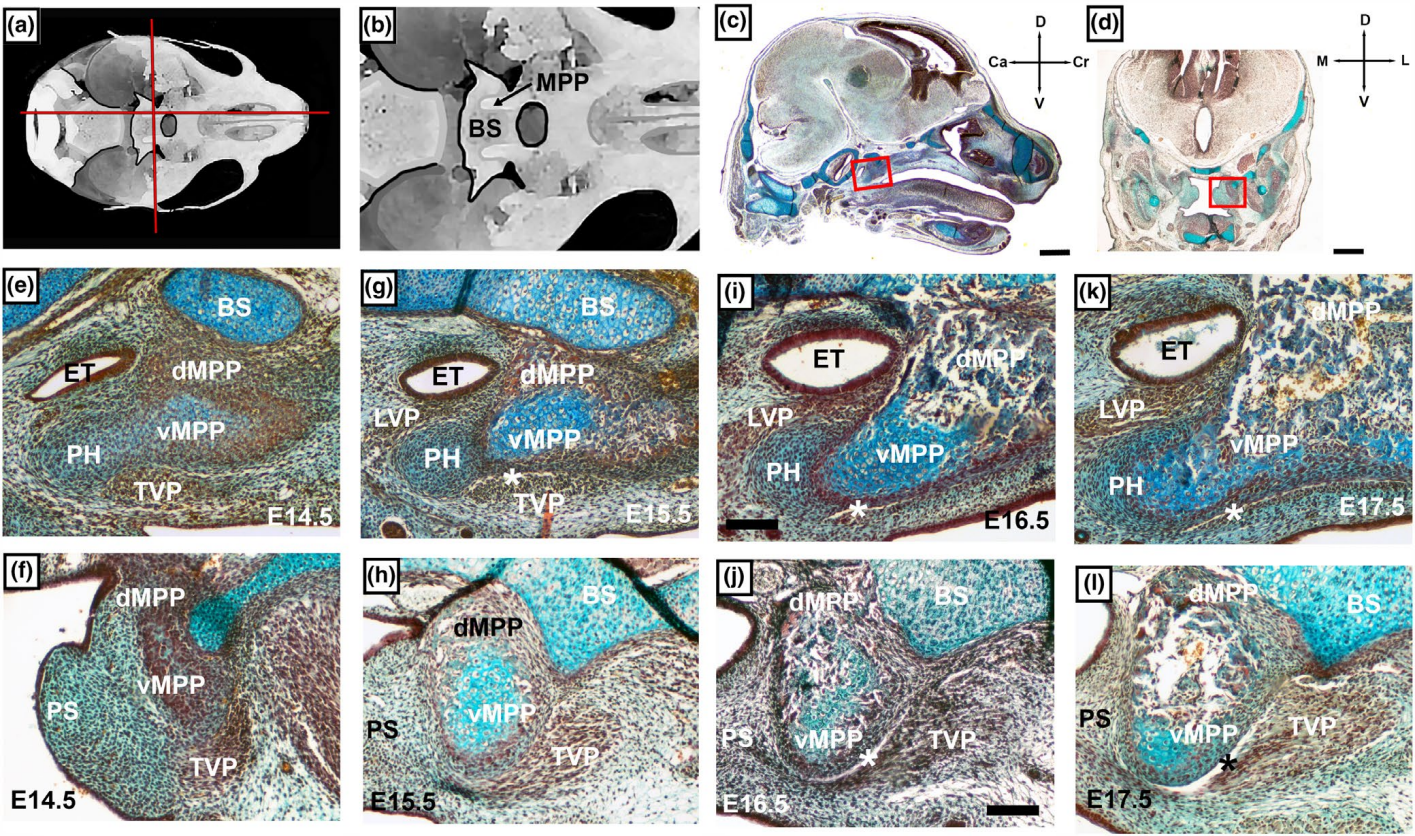
Development of the murine medial pterygoid plate. (a, b) Schematic of mouse palatal region from skeletal prep at E18.5. (a) Red lines show sagittal and frontal plane of section shown throughout paper. (b) Medial pterygoid plate (MPP) and Basispenoid. (c–l) Trichrome‐stained sections from wildtype mice aged E14.5–E17.5 in both sagittal (c, e, g, i, k) and frontal (d, f, h, j, l) section. Alcian blue (blue), Sirius red (red) and haematoxylin (purple). (c) Trichrome of an E14.5 mouse head in sagittal section highlighting the position of the pterygoid by the red rectangle. Axes: D, dorsal; Cr, cranial; V, ventral; Ca, caudal. (d) Trichrome of an E14.5 mouse head in frontal orientation, with the position of the pterygoid highlighted by a red rectangle. Axes: D, dorsal; V, ventral; M, medial; L, lateral. (e, f) At E14.5, condensations of both the medial pterygoid plate and the hamulus are visible within the mesenchyme, and the TVP has started to move under the medial pterygoid during palatal shelf elevation. (g, h) By E15.5, the dorsal medial pterygoid plate has fully ossified. A bony collar has started to form around the ventral medial pterygoid, while a bursa develops between the plate and TVP *. (i, j) At E16.5, the medial pterygoid has differentiated further and started to fuse with the basisphenoid (BS). (k, l) At E17.5, ossification extends ventrally into the ventral medial pterygoid plate. *, bursa formation; BS, basisphenoid; dMPP, dorsal medial pterygoid plate; ET, Eustachian tube (pharyngotympanic tube); LVP, Levata veli palatini; PH, pterygoid hamulus; PS, palatal shelves; TVP, Tensa veli palatini; vMPP, ventral medial pterygoid. Scale bars: c, d = 500 μm; j = 100 μm (same scale in e–l).

All tissues for histological sectioning were fixed overnight at 4°C in 4% paraformaldehyde (PFA). Samples were then dehydrated through a series of graded ethanol and cleared with Neoclear before infiltration with paraffin wax at 60°C. Wax‐embedded samples were sectioned with a microtome at 8 μm thickness in the frontal and sagittal planes (see Figure 1a,b). The samples were then mounted in a parallel series on charged slides and left to dry. For orientation of structures within the head, we used cranial, caudal, dorsal, and ventral as standardly used for quadrupeds in veterinary science and animal models (Dyce & Sack, 2009) (see axes in Figure 1c,d). Dorsal refers to superior and ventral to inferior in the homologous structures in Humans.

### 2.3 Trichrome staining

For histological examination of the bones and cartilage, serial sections of the pterygoid and soft palate were stained with 0.5% Sirius red, Alcian blue, and Ehrlich's Haematoxylin. The slides were then mounted using Neomount and covered with a glass coverslip. The Nikon Eclipse 80i light microscope was used to examine and capture the trichrome‐stained slides.

### 2.4 Immunofluorescence

Immunohistochemical staining was conducted by first deparaffinising with Neoclear before rehydrating through a graded ethanol series to PBS. Heat‐induced antigen retrieval was conducted by placing the samples into a 95°C water bath for 45 min in 0.1 M sodium citrate pH 6 buffer. Slides were then blocked with a solution of PBS, 0.025% Tween 20, 10% donkey serum, and 1% Bovine serum albumin for 1 h at room temperature. These samples were then treated overnight at 4°C with primary antibodies. The primary antibodies used were as follows: goat anti‐sox9, rabbit anti‐runx2, mouse anti‐12/101, goat anti‐RFP, rabbit anti‐Sox9, and rat anti‐RFP. Information regarding dilutions and product codes for the primary antibodies can be found in Table S1. Secondary antibodies were added after repeated washes in PBS. For fluorescent labelling, the following secondary antibodies were used at a dilution of 1/300: 488 nm donkey anti‐goat, 568 nm donkey anti‐rabbit, 647 nm donkey anti‐mouse, 568 nm donkey anti‐goat, 647 nm donkey anti‐rabbit, and 647 nm donkey anti‐rat (all Invitrogen). Secondary antibodies were added in the blocking buffer for 1 h at room temperature in the dark. The secondary antibody was then washed off with PBS, and the slides were mounted using Fluoroshield medium with DAPI (Abcam). Sections were then visualised using a Zeiss Apotome at the CCRB and the inverted confocal fluorescence microscope at the Nikon Imaging Centre.

### 2.5 Collagen hybridising peptide staining

Tissue sections were deparaffinised and rehydrated. 5% Goat serum in PBS was added and incubated for 30 min at RT to block nonspecific binding. The biotin‐conjugated collagen hybridising peptide (B‐CHP) solution (15 μM, 50 μL per section) was made up in 1% BSA. The solution was heated in the oven at 80°C for 5 min and immediately incubated on ice for 15 s before application. Slides were incubated with B‐CHP solution overnight at 4°C (Hwang et al., 2017). The next day, slides were treated with Alexa Fluor® 647‐streptavidin solution (0.005 mg/mL) in 1% BSA for 1 h at RT. Nuclear counterstaining was performed using DAPI.

## 3 RESULTS

### 3.1 Multiple elements unite to create the medial pterygoid plate

To investigate the normal development of the medial pterygoid plate, frontal and sagittal histological sections were analysed from embryonic day E14.5 to E17.5. For orientation, sections show approximate region highlighted by the boxes in Figure 1c,d. At E14.5, the medial pterygoid plate (MPP) was evident as two distinct condensations: a dorsal anlage (dMPP), which stained faintly with sirius red and was positioned under the cartilaginous basisphenoid (BS), and a ventral condensation (vMPP) stained with alcian blue (Figure 1e,f). By E15.5, the medial pterygoid plate displayed a distinct bipartite composition: the dMPP stained strongly with sirius red and formed a bridge between the cartilaginous basisphenoid and vMPP. The pterygoid hamulus (PH) was prominent, appearing as a rounded structure situated at the posterior edge of the medial pterygoid (Figure 1g,h). At E16.5, the dMPP underwent ossification and had started to fuse with the ventral aspect of the basisphenoid, which remained cartilaginous (Figure 1i,j). At E16.5 and E17.5, the pterygoid hamulus appeared as a cap around the vMPP and extended anteriorly along the ventral edge of the MPP (Figure 1i,k). By E17.5, the medial pterygoid plate had fused to the basiphenoid (Figure 1k,l). In parallel with the development of these structures, a synovial bursa formed under the medial pterygoid plate at the position of the Tensor Veli Palatini (TVP). This bursa functions as a friction‐reducing interface as the TVP contracts and relaxes the soft palate. Bursa formation was evident as a slight crack as early as E14.5, with the cracks extending and becoming more defined during the cavitation process from E15.5 to E17.5 (Figure 1g–l).

### 3.2 Distinct molecular signatures of the medial pterygoid plate

**FIGURE 2:**
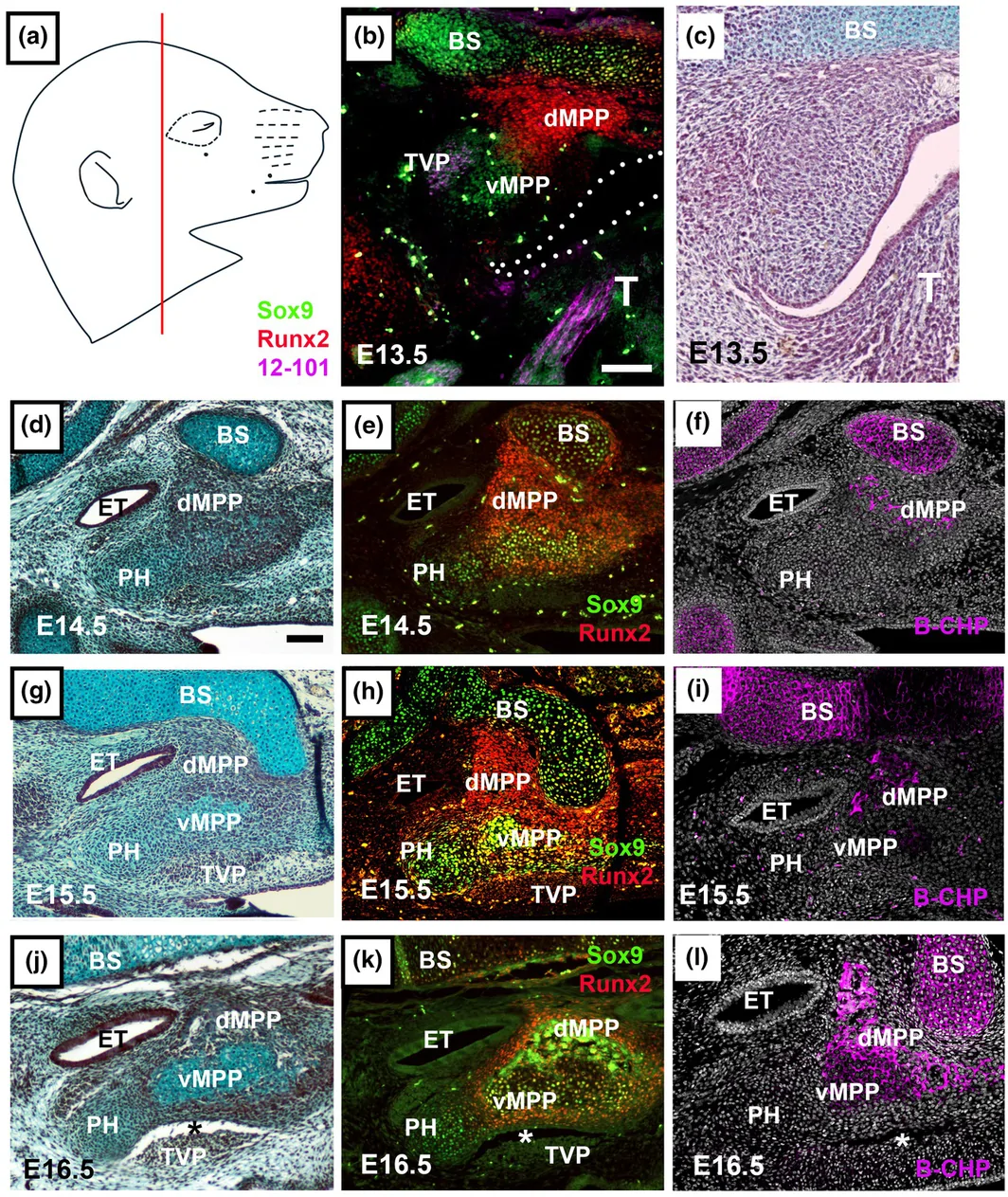
Distinct modes of development of the medial pterygoid plate. (a–c) Frontal section E13.5. (d–l) Sagittal section E14.5–16.5. Immunofluorescence patterns in wildtype mice aged E13.5–E16.5 using antibodies against Sox9 (green), Runx2 (red), and 12/101 (magenta in b). Collagen remodelling shown in magenta in (f, i, l) by B‐CHP. (a) Schematic of a mouse embryo showing the plane of the frontal section in (b, c). (b) Frontal section of an E13.5 embryo demonstrating the two distinct condensations of the dorsal (Runx2‐positive) and ventral (Sox9‐positive) medial pterygoid plate and the position of the tensor veli palatini (TVP). (c) Serial section stained for trichrome. (d) Trichrome‐stained section of an E14.5 mouse. (e) Serial section, IF for Sox9 and Runx2. Runx2 expression is found in the dorsal medial pterygoid plate and has extended into the ventral medial pterygoid plate. (f) Serial section, B‐CHP staining to show pattern of collagen remodelling. (g) Trichrome‐stained section of an E15.5 mouse. (h) Serial section, IF for Sox9 and Runx2 showing co‐expression in the ventral medial pterygoid as this plate undergoes endochondral ossification. The hamulus remains Sox9 positive only. (i) B‐CHP staining in an equivalent section showing the pattern of collagen remodelling. (j) Trichrome‐stained section of an E16.5 wildtype mouse. (k) Serial section, IF for Sox9 and Runx2. The dorsal medial pterygoid has started to ossify, indicated by tissue autofluorescence (green in bone matrix of dVPP). A bursa (*) is evident between the medial pterygoid plate and the TVP muscle. (l) B‐CHP staining in an equivalent section showing collagen remodelling extending into the vMPP, but not the hamulus. *, bursa formation; BS, basisphenoid; dMPP, dorsal medial pterygoid plate; ET, Eustachian tube; PH, pterygoid hamulus; T, tongue; TVP, tensor veli palatini; vMPP, ventral medial pterygoid plate. Scale bar in (b) and (d) 100 μm. Same scale as in (d) in (e–l).

Endochondral and intramembranous ossification are the two primary processes by which bones form in vertebrates. In endochondral ossification, mesenchymal tissue first differentiates into cartilage, which is subsequently replaced by bone (Šromová et al., 2023). In contrast, intramembranous ossification involves the direct transformation of mesenchymal tissue into bone, bypassing a cartilaginous intermediate. To confirm the different modes of development of the medial pterygoid plate, expression of Runx2 (a marker of osteogenesis and hypertrophic cartilage) and Sox9 (a marker of chondrogenesis) was analysed from E13.5 to E16.5 (Figure 2). At E13.5, expression of Runx2 and Sox9 was already detectable in frontal sections via immunofluorescence, before the medial pterygoid was evident by histology (Figure 2a–c). Sox9 was strongly expressed in the basisphenoid, which stained for alcian blue (Figure 2b,c). In contrast, the dorsal MPP showed robust expression of Runx2, confirming its origin as a membranous bone. Under the dMPP, the vMPP has started to express Sox9, but did not yet stain for alcian blue (Figure 2b,c). This agrees with previous descriptions of the vMPP as a late‐developing secondary cartilage (i.e. forms after the first wave of bone formation has started). The TVP, as stained with the muscle marker 12–101, was positioned next to the forming vMPP (Figure 2b). The vMPP and dMPP, therefore, have distinct markers in early development.

As the murine medial pterygoid curves caudally, we then shifted to sagittal sections to view the medial pterygoid plate and hamulus more clearly (see Figure 1a,c) for orientation. From E14.5 to E16.5, the pterygoid hamulus expressed Sox9, with no evidence of Runx2 (Figure 2e,h,k). The murine pterygoid hamulus only ossified postnatally at weaning (P21) (data not shown). The initially Sox9‐positive basisphenoid and the ventral MPP started turning on Runx2 from E15.5 as the cartilage underwent hypertrophy during endochondral ossification (Figure 2g,h). The dorsal MPP continued to express Runx2 only. By E16.5, a distinct border could be observed between the Sox9‐positive hamulus and the Sox9/Runx2 positive vMPP (Figure 2j,k). Collagens are a significant matrix for cartilage and bone and undergo constant remodelling during development. To follow collagen remodelling during the development of the pterygoid, we utilised biotin‐conjugated collagen hybridising peptide (B‐CHP), which binds to unfolded/denatured collagen chains. At E14.5 collagen remodelling was evident in the cartilaginous basisphenoid and the dorsal MPP but was absent from the vMPP and hamulus (Figure 2f). By E15.5, collagen remodelling had reduced in the part of the basisphenoid that had started endochondral ossification but was still found in the dMPP (Figure 2i). By E16.5 and E17.5 collagen remodelling had spread into the vMPP but was excluded from the cartilaginous pterygoid hamulus (Figure 2l; Figure S1A). The timing and level of collagen remodelling in the pterygoid was, therefore, element specific, reflecting the different modes and stages of development.

### 3.3 The medial pterygoid plate and hamulus is of neural crest origin

**FIGURE 3:**
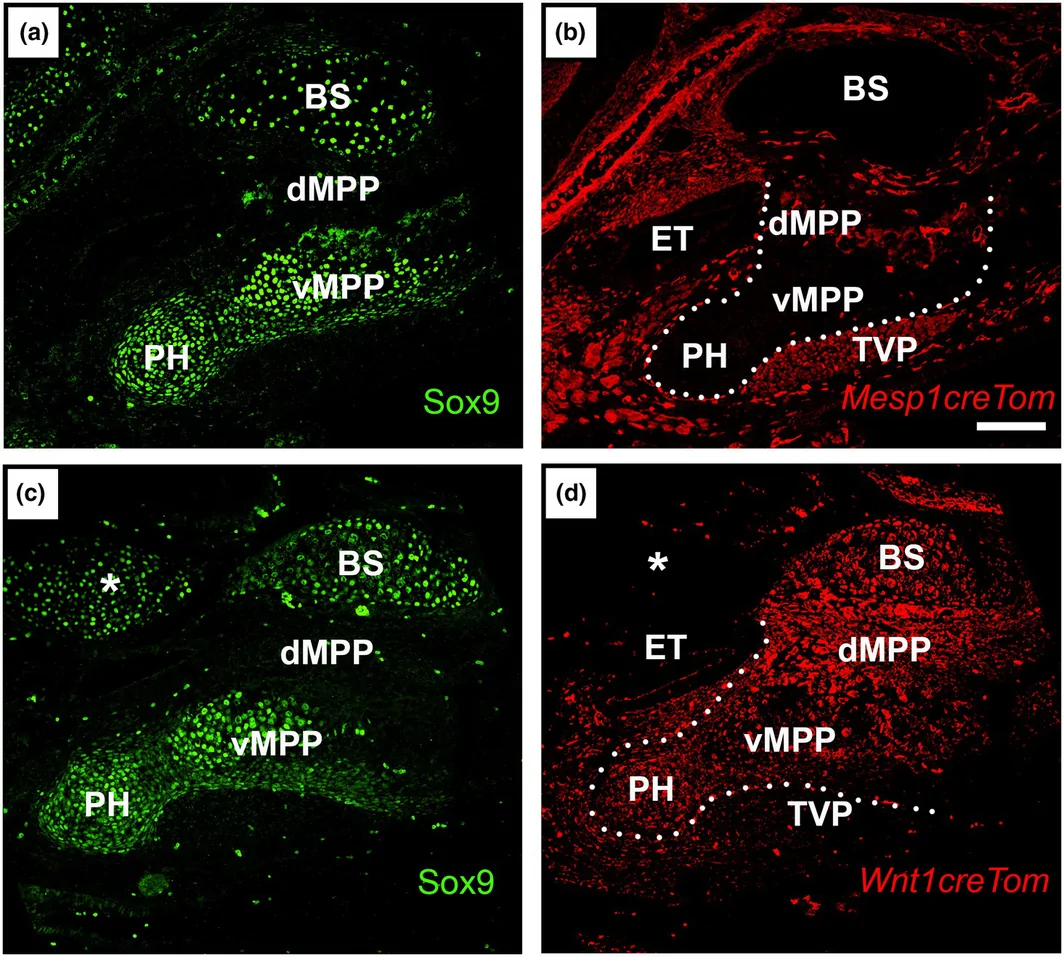
Lineage tracing of the medial pterygoid plate. (a, b) E15.5 *Mesp1Cre;tdTom*. (c, d) E15.5 *Wnt1Cre;tdTom* reporter mice. Sagittal sections. (a) Sox9 expression (green) in *Mesp1Cre;tdTom* showing cartilage in the basisphenoid, ventral medial pterygoid plates, and the hamulus. (b) Serial section showing RFP expression in *Mesp1Cre;tdTom*, marking myogenic mesodermal tissue in red. Expression in the dorsal medial pterygoid plate is invading vasculature. The basisphenoid above the medial pterygoid is not labelled. (c) Sox9 expression (green) in *Wnt1Cre;tdTom*. (d) Serial section showing RFP expression in *Wnt1Cre;tdTom* (red). Consistent label shown throughout the medial pterygoid plate and overlying basisphenoid. The fusion site of the basisphenoid was labelled, but the more caudal part of the sphenoid was not derived from the neural crest (asterisk). Dotted lines in (b) and (d) outline the medial pterygoid plate. BS, basisphenoid; dMPP, dorsal medial pterygoid plate; ET, Eustachian tube; PH, pterygoid hamulus; TVP, Tensor veli palatini; vMPP, ventral medial pterygoid plate. Scale bars in (b) 100 μm (same scale in a, c, d).

To determine the embryonic origin of the medial pterygoid plate, lineage tracing using *Mesp1Cre;tdTom* and *Wnt1Cre;tdTom* reporter lines was performed to label mesoderm and neural crest‐derived cells, respectively at E15.5. Expression of Sox9 was used to outline the basisphenoid, vMPP, and hamulus in serial sections (Figure 3a,c). All elements of the medial pterygoid plate were largely negative for red fluorescent protein (RFP) in the *Mesp1Cre;tdTom* reporter mice, which labelled the mesoderm‐derived muscles and vasculature (Figure 3b). A domain in the ossifying part of the dMPP showed labelling, but this consisted of autofluorescence from the forming bone matrix and the influx of mesoderm‐derived endothelial cells, which play an important role during ossification (Figure S2). In contrast, in the *Wnt1Cre;tdTom* line, the entire medial pterygoid plate, hamulus, and adjacent basisphenoid were labelled with RFP (Figure 3d). Interestingly, the more caudal part of the basisphenoid further from the medial pterygoid plate was not labelled in the *Wnt1Cre;tdTom* mice, suggesting that this region represents the border between the neural crest and mesoderm (Figure 3d). This data confirms that neural crest‐derived mesenchyme contributes extensively to the formation of the medial pterygoid plate.

### 3.4 The vMPP and hamulus guide early muscle pathfinding

**FIGURE 4:**
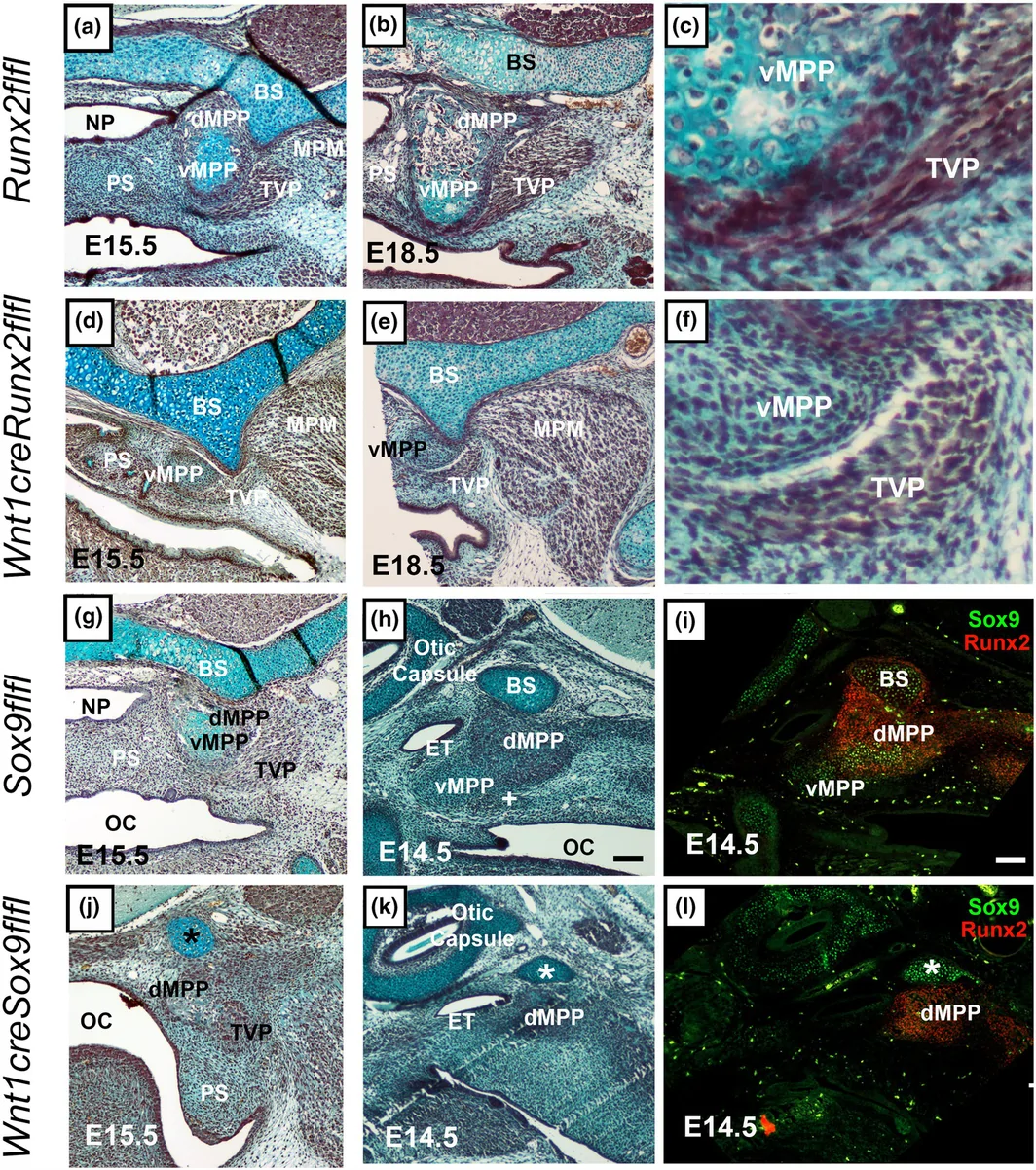
Muscle defects after loss of the cartilage components of the medial pterygoid plate. (a–f, g, j) Frontal sections. (h, i, k, l) Sagittal sections. (a–c) Littermate controls *Runx2* ^*fl/fl*^. (d–f) *Wnt1Cre;Runx2* ^*fl/fl*^. (a) Section showing the TVP under the medial pterygoid towards the palatine aponeurosis at E15.5. (b) Similar section at E18.5. (c) High power of (b) showing TVP and vMPP. (d) *Wnt1Cre;Runx2* ^*fl/fl*^ section showing loss of the dorsal membranous bone, leaving the ventral endochondral medial pterygoid and hamulus intact at E15.5. (e) Similar section at E18.5. (f) High power of (e) showing TVP and vMPP. (g) Frontal section showing the close association of the ventral medial pterygoid and TVP at E15.5 *Sox9* ^*fl/fl*^. (h) Sagittal section showing the close association of the ventral medial pterygoid and TVP (marked by +) at E14.5 in *Sox9* ^*fl/fl*^ littermate. (i) Serial section showing Sox9 (green) and Runx2 (red). (j) *Wnt1Cre;Sox9* ^*fl/fl*^ frontal section showing an intact dorsal medial pterygoid plate and residual mesodermal cartilage of the sphenoid (*) at E15.5. (k) Sagittal section showing loss of the ventral medial pterygoid and hamulus and absence of the TVP in this plane at E14.5 in *Wnt1Cre;Sox9* ^*fl/fl*^. (l) Serial section showing Sox9 (green) and Runx2 (red). No evidence of the ventral Medial pterygoid or hamulus in the mutant. *, mesoderm‐derived cartilage; BS, basisphenoid; dMPP, dorsal medial pterygoid plate; ET, Eustachian tube; MPM, medial pterygoid muscle; NP, nasopharynx; OC, oral cavity; PS, palatal shelves; TVP, tensor veli palatini; vMPP, ventral medial pterygoid plate. Scale bar in (h) 100 μm, same scale in (a, b, d, e, g–l).

Given the dual nature of the medial pterygoid plate, and its relationship to the TVP we investigated how loss of one part of the plate might impact the rest of the structure. For this we analysed *Wnt1Cre; Runx2* ^*fl/fl*^ and *Wnt1Cre; Sox9* ^*fl/fl*^ conditional knockout embryos. To view the TVP wrapping under the pterygoid hamulus we moved to frontal sections to follow the path of the TVP towards the aponeurosis. In the *Wnt1Cre; Runx2* ^*fl/fl*^ embryos, the dorsal component of the medial pterygoid failed to form, confirming that *Runx2* was required for the intramembranous part of the medial pterygoid (Figure 4a,b,d,e). The cartilages of the basisphenoid and ventral medial pterygoid still formed as expected but did not undergo hypertrophy at later stages (Figure 4b,e). Runx2 has been shown to be expressed in the perimysial population of the LVP but not the TVP (Han et al., 2021). Interestingly, the TVP could be observed wrapping around the cartilaginous ventral medial pterygoid in the *Wnt1Cre; Runx2* ^*fl/fl*^, highlighting that the superior part of the medial pterygoid was not required for the correct patterning of this muscle (Figure 4c,f). Interestingly, the TVP still wrapped under the cartilage despite a cleft palate being present in these mutants.

In the *Wnt1Cre; Sox9* ^*fl/fl*^ embryos, chondrogenesis was disrupted in the neural crest‐derived elements, but the dorsal medial pterygoid ossified as normal (Figure 4g–l). A small cartilage was observed in the region of the basisphenoid (marked by an *) (Figure 4j–l), agreeing with the lineage tracing that suggests that the basisphenoid is positioned at the border between the neural crest and mesoderm (Figure 3d). In these embryos, the TVP could not pathfind and remained amorphous alongside the isolated dorsal MPP (Figure 4j). A cleft palate was also present in these mutants. Analysis of mutants in sagittal section with Sox9 and Runx2 IF confirmed the loss of the ventral MPP and hamulus and neural crest‐derived part of the basisphenoid (Figure 4i,l). Similar to frontal sections, sagittal sections confirmed that the TVP had not migrated under the pterygoid in the absence of the cartilaginous elements (Figure 4h,k).

## 4 DISCUSSION

### 4.1 The medial pterygoid plate and pterygoid hamulus develop in a coordinated, bipartite manner

The findings presented here provide new insights into the ontogeny and structural integration of the pterygoid process within the mammalian skull, and its relationship with the tensor veli palatini (TVP) muscle during embryogenesis. Our results extend previous work based on histology stains (Hirouchi et al., 2018; Yamamoto et al., 2017) and confirm that the medial pterygoid plate is a compound structure, with distinct endochondral (Sox9‐positive) and intramembranous (Runx2‐positive) components. The dorsal medial pterygoid plate (MPP) underwent Runx2‐dependent membranous ossification, while the ventral medial pterygoid plate followed a Sox9‐dependent endochondral programme. These differences likely reflect divergent signalling environments and mechanical demands. The membranous dorsal MPP and the primary cartilage of the basisphenoid underwent high levels of collagen remodelling at E14.5 onwards, while the cartilage of the vMPP underwent remodelling later, consistent with the classification of the ventral MPP as a late‐developing secondary cartilage (Hirouchi et al., 2018). The emergence of the pterygoid hamulus as a ball‐like structure at the end of the vMPP suggested it forms as a separate entity and was not simply an extension of the medial pterygoid plate (Yamamoto et al., 2017), agreeing with older interpretations of it as an independent structure (Edgeworth, 1914) and analysis from human embryos (De la Cuadra Bnco et al., 2012). The hamulus remained cartilaginous throughout mouse embryonic development and did not undergo collagen remodelling during this time period.

### 4.2 Neural crest derivation of the entire pterygoid process

Lineage tracing confirmed that the medial pterygoid process, hamulus, and associated region of the basisphenoid was of neural crest origin. Our findings align with those of McBratney‐Owen et al. (2008), who similarly traced neural crest contributions to the sphenoid and pterygoid regions. In contrast, Shirai et al. (2019) reported some mesodermal input. However, our use of *Mesp1Cre* and *Wnt1Cre* lines showed no evidence of mesodermal contribution to the pterygoid, although some of the basisphenoid was of mesoderm origin, consistent with the medial pterygoid plate being located at the neural crest‐mesoderm boundary zone described by Evans and Noden (2006). These discrepancies may arise from technical differences in lineage marker sensitivity or resolution. We did observe some mesodermally derived tissue in the medial pterygoid plate, but these cells had the morphology of endothelial cells which had invaded to take part in ossification. Our results, therefore, provide strong support for a purely neural crest origin.

### 4.3 Functional specialisation confirmed by Runx2 and Sox9 knockouts

Conditional deletion of *Runx2* and *Sox9* in neural crest‐derived lineages further elucidated the ossification dynamics of the medial pterygoid plate. Loss of *Runx2* in the neural crest abolished formation of the dorsal medial pterygoid plate, which confirmed that this component develops through membranous ossification. In contrast, loss of *Sox9* in the neural crest disrupted chondrogenesis in the ventral medial pterygoid and hamulus, confirming its essential role in endochondral ossification (Šromová et al., 2023). These findings mirror established roles of *Runx2* and *Sox9* in craniofacial development (Akiyama et al., 2002).

### 4.4 Muscle pathfinding of the tensor Veli palatini

The tensor veli palatini (TVP) was evident at E13.5 positioned next to the Sox9‐positive vMPP and had already wrapped under the vMPP by E14.5. At these stages, rhythmic jaw and tongue movements have started to initiate in the embryo, linking spontaneous foetal motor activity to the mechanical architecture required for suckling (Tsunekawa et al., 2005). Interestingly in *Wnt1cre;Runx2* mutants, the TVP still wrapped around the remaining cartilage, suggesting that cartilage may be the primary scaffold for early TVP guidance. The TVP also was shown to wrap around the medial pterygoid plate in *Osr2cre;Runx2* mutants, where the medial pterygoid plate was reduced (Han et al., 2021). This arrangement of the TVP was evident even in the presence of a cleft palate, in keeping with the finding that the TVP was normal, but the aponeurosis was absent, in human foetuses with clefts (Ross, 1971). In contrast, in *Sox9* mutants, where the vMPP and hamulus were missing, the TVP lost its trajectory and remained lateral to the pterygoid. Taken together, these results show that the ventral endochondral MPP and hamulus provide the critical structure to direct the migration of the TVP muscle.

## 5 CONCLUSIONS

The medial pterygoid plate and the pterygoid hamulus represent a dynamic craniofacial structure whose development involves a coordinated interplay of intramembranous and endochondral ossification processes, as well as the formation of secondary cartilage along with the fusion of the medial plate with the rest of the sphenoid. Our findings, supported by lineage tracing and conditional knockout models, highlight the distinct molecular regulation of ossification and chondrogenesis within these components and highlight their importance for patterning the surrounding musculature. In the future, it will be interesting to assess whether the muscle influences the development of the pterygoid hamulus and medial pterygoid plate and to understand the mechanisms that may have led to the evolution of this complex structure during mammalian evolution.

## AUTHOR CONTRIBUTIONS

AST conceived the idea and acquired funding. LAB and EN performed the histology and immunofluorescence experiments. LAB and AST wrote the manuscript. All authors contributed to the article and approved the submitted version.

## FUNDING INFORMATION

LAB was funded by the Anatomical Society from a PhD Studentship awarded to AST. A JASSO scholarship supported EN during their visit to King's College London.

## CONFLICT OF INTEREST STATEMENT

Abigail Tucker is the President of the Anatomical Society.

## Supporting information


Figures S1–S2.

## Data Availability

The data that supports the findings of this study are available in the supplementary material of this article.
