# Supplementary material for: Secondary cartilage in the murine medial pterygoid plate has a critical role in the pathfinding of the tensor veli palatini
Source: J Anat. 2026 Mar 15;249(4):759–68. doi: 10.1111/joa.70133 (PMC13398515; doi:10.1111/joa.70133)
Supplement: Supplementary file 1 [file JOA-249-759-s001.docx]

Supplementary data:

**Supplementary table**

| **Primary Antibody** | **Raised in** | **Company** | **Primary**  **Product Number** | **Primary antibody**  **dilution** | **Secondary Antibody** |
| --- | --- | --- | --- | --- | --- |
| Anti-Sox9 | Goat | R & D systems | AF3075 | 1/200 | Donkey anti-goat 488nm |
| Anti-Runx2 | Rabbit | Abcam | AB192256 | 1/1000 | Donkey anti-rabbit 568nm |
| Anti-12/101 | Mouse | Developmental Studies Hybridoma Bank | DSHB 12/101 | 1/50 | Donkey anti-mouse 647nm |
| Anti-RFP | Goat | Antibodies.com | A121671 | 1/300 | Donkey anti-goat 568nm |
| Anti-Sox9 | Rabbit | Merck Millipore | AB5535 | 1/200 | Donkey anti-rabbit 647nm |
| Anti-RFP | Rat | Chromotek | 5F8 | 1/300 | Donkey anti-rat 647nm |

Supplementary Figure 1: Collagen remodelling at E17.5 in the murine medial pterygoid plate

(A) B-CHP staining showing collagen remodelling at E17.5. B-CHP in magenta. DAPI white. Medial pterygoid outlined with white dots. PH = Pterygoid hamulus, vMPP = ventral medial pterygoid process, dMMP = dorsal medial pterygoid process.

Supplementary Figure 2: Mesoderm contributes to the invading vasculature during ossification of the medial Pterygoid plate

(A,B) Trichrome staining Sagittal E15.5 in *Mesp1creTdTom* mice. Serial sections labelled with Sox9 (C,D) and RFP (Red fluorescent protein)(E,F). (C,D) Sox9 labels the cartilaginous elements of the medial pterygoid plate. (D,F) Bone matrix autofluoresces in both the green and red channels (see asterix). (F) The developing bone is associated with invading vasculature which is mesoderm derived (see cross). BS: basispenoid, vMPP: ventral medial pterygoid plate, dMPP: dorsal medial pterygoid plate. ET: Eustachian tube.
